# A magnifying glass onto renal function and serum lipid evolutions after tenofovir (TDF) and emtricitabine (FTC) in combination with atazanavir/ritonavir (ATV/r) versus efavirenz (EFV) as first-line HAART (the INCA trial)

**DOI:** 10.1186/1758-2652-13-S4-P85

**Published:** 2010-11-08

**Authors:** I Izzo, L Albini, A Calabresi, D Motta, R Bellagamba, R Fezza, P Narciso, L Sighinolfi, P Maggi, E Focà, M Mendeni, L Manili, M Magoni, G Carosi, E Quiros-Roldan, C Torti

**Affiliations:** 1 Institute of Infectious and Tropical Diseases, University of Brescia, Brescia, Italy; 2 National Institute of Infectious Diseases, Rome, Italy; 3 “S. Anna” Hospital, Ferrara, Italy; 4 Department of Nephrology, Spedali Civili, Brescia, Italy;; 5 Chair of Hygiene, University of Brescia, Brescia, Italy

## Background

Measures of glomerular filtration rate (GFR) showed discordant results. CKD-EPI creatinine formula resulted more accurate than other equations in subjects with normal or mildly decreased renal function in the general population and cystatin C could be even a more sensitive measure. As for lipids, lipoprotein subfractions were suggested to be more informative on the cardiovascular risk than the commonly used cholesterol subfractions, with protection conferred especially by HDLp-small (Baker et al. JID 2010). Prospective evaluation of all these markers upon initiation of standard HAART regimens is lacking. We conducted a small, intensive, randomized study in naïve HIV-infected patients comparing TDF/FTC+ATV/r versus TDF/FTC+EFV as first-line therapy for these novel markers.

## Methods

Antiretroviral-naïve HIV-infected patients, recruited from 4 centers in Italy (Brescia, Rome, Ferrara, Bari), were randomized to ATV/r or EFV standard doses, in combination with fixed-dose TDF/FTC. Patients had to have creatinine clearance>50 ml/min. Outcome measures included serum creatinine and cystatin C levels and derived eGFRs corrected for body surface area. Lipoprotein particle size and concentration were estimated using an NMR spectroscopy method at Jochen-Hunter Lab., Germany.

## Results

91 patients were randomized (48 ATV/r, 43 EFV; 80% males; mean age 43 years; 4 patients class C; mean CD4+ 283/mm3, SD: 119/mm3). No significant differences were found between the two arms at baseline, but for some lipids (total cholesterol: mean 173 mg/dL ATV/r vs. 156 mg/dL EFV; p=0.04; HDL-cholesterol: 44 mg/dL vs. 38 mg/dL; p=0.007; HDLp: 36 mg/dL vs. 32 mg/dL; p=0.02). At baseline, a correlation between CKD-EPI creatinine and CKD-EPI cystatine C was found (R2=0.51; p<0.0001). Through CKD-EPI creatinine formula, we detected a significant decrease in eGFR from baseline to week 48 in patients, receiving ATV/r (-4.8233 mL/min/m2; p=0.002) but not in those receiving EFV. Greater GFR reductions were found with CKD-EPI cystatin C than with CKD-EPI creatinine not only in the ATV/r arm up to week 48 (-15.1388 mL/min/m2; p<0.0001), but also in the EFV arm from baseline to week 24 (-7.2233 mL/min/m2; p=0.04). As for lipids, cholesterol subfractions increased more after EFV than after ATV/r: mean increase from baseline to week 48 was 44.15 mg/dL vs. 15.51; p=0.002 for total cholesterol, 32.4 mg/dL vs. 12.47 mg/dL; p=0.007 for LDL cholesterol, 9.31 mg/dL vs. 2.13 mg/dL; p=0.002 for HDL cholesterol. Total/HDL cholesterol ratio remained stable in both arms. As for lipoprotein subfractions, total HDLp increased more after EFV than after ATV/r (11.97 mg/dL vs. 7.97 mg/dL; p=0.03), but HDLp-small increased significantly in both arms, without statistical differences between the two (1.71 mg/dL vs. 1.86 mg/dL).

## Conclusions

Patients receiving TDF in combination with ATV/r had greater decline in renal function than those receiving TDF plus EFV, although eGFR decrease was small in both arms. Interestingly, CKD-EPI cystatin C appeared to be a stricter measure. As for lipids, EFV induced greater LDL cholesterol increases but the risk appeared to be counteracted by greater increase of both HDL-cholesterol and HDLp, even though HDLp-small increased similarly in the two arms. We suggest that these novel measures provide additional information so as to better characterize the toxicity profiles of the antiretroviral regimens.

